# Causes of death in children and adolescents aged 1-19 in Poland in the light of international statistics since 2000

**DOI:** 10.34763/devperiodmed.20172102.111123

**Published:** 2017-08-11

**Authors:** Joanna Mazur, Marta Malinowska-Cieślik, Anna Oblacińska

**Affiliations:** 1 Zakład Zdrowia Dzieci i Młodzieży, Instytut Matki i Dziecka, Warszawa, Polska; 2 Instytut Zdrowia Publicznego, Wydział Nauk o Zdrowiu, Uniwersytet Jagielloński Collegium Medicum, Kraków, Polska

**Keywords:** umieralność, przyczyny zgonów, zgony możliwe do uniknięcia, trendy, porównania międzynarodowe, mortality, causes of death, avoidable mortality, trends, international comparisons

## Abstract

**Background:**

Analyses of children and young people mortality continue to be an important component of health monitoring of this population. Such analyses provide the basis to assess the overall trends, the structure of the causes of death over longer periods, and the differences between Poland and other countries.

**Purpose:**

The purpose of the current study is to present the current status and the direction of changes since 2000 with regard to the level and underlying causes of mortality in children and adolescents aged 1-19 years in Poland on the background of statistics for leading European countries.

**Material and methods:**

Interactive databases available online: the National Demographic Database provided by the Central Statistical Office and the International WHO-MDB Database were used. Poland, constantly belonging to Eur-B category, was compared with the combined group of 27 leading countries, classified as a very low total mortality group (Eur-A) according to WHO. Linear trends of overall and cause-specific mortality in 2000–2013 were estimated. The causes of death have been presented according to the main classes of the 10th Revision of the International Statistical Classification of Diseases and Related Health Problems (ICD-10). External and other causes were adopted as the two principal categories.

**Results:**

In 2015, 1471 deaths of persons aged 1-19 were recorded in Poland (19.9 per 100 000, 25.4 and 14.2 for boys and girls, respectively). Changes in children and adolescents mortality by age have a nonlinear nature (U-shaped), and the lowest level is recorded at the age of 5-9 years.

According to 2014 data, 50.2% of deaths of children and adolescents aged 1-19 years occurred due to external causes, including non-intentional and intentional ones. This percentage increased from 18.4% in the 1-4 age group to 68.6% at the age of 15-19 years. Apart from external causes, the dominating causes of death are malignant neoplasms, congenital defects, or nervous system and respiratory system diseases. The ranking of those causes of death changes in successive age groups and over time. When age is considered, a higher proportion of congenital defects and respiratory system diseases was found in mortality younger children and a higher proportion of circulatory system diseases and undefined cases in mortality of adolescents. When trends were studied, a continuing elimination of infectious diseases was observed together with growing impact of rare diseases in all age groups.

The excess mortality of Polish population at age 1-19 by comparison to Eur-A countries increased from 21% in 2000 to 56% in 2013, mainly due to unfavourable trends in adolescents. The rate of decline in the mortality of young children (1-4 years) was greater than in Eur-A countries, both in case of external and other causes. In the age group 5-14 years the higher rate of change was sustained only with regard to external causes. Among adolescents and young adults, the distance between Poland and Eur-A countries increased during the studied period. The shape of trend in the 15-24 age group was unfavourable for Poland, mainly with respect to external causes. This observation could be in part explained by increasing suicide trend in Poland since 2008, coexisting with rather constant level in Eur-A countries.

**Conclusions:**

The mortality rate among the population aged 1-19 years in Poland is systematically decreasing, but it still exceeds the average level recorded in leading European countries, particularly in relation to adolescents. When assessing the ability to reduce mortality in Poland to the level of Eur-A countries, attention must be paid to the causes considered as avoidable. Further studies ought to focus on the trends and international comparisons only foreshadowed in this study with regard to individual diagnoses, discussing possible preventive measures. Introduction of an ICD-11 classification will enable more accurate coding of causes of death, including a more precise analysis of the burden of rare diseases, which are an increasing challenge to public health in the population at the developmental age.

## Streszczenie

**Tło badań:**

Analizy umieralności dzieci i młodzieży są ciągle ważnym elementem monitorowania stanu zdrowia tej populacji. Poprzez systematyczne ich powtarzanie można wykazać różnice w strukturze przyczyn zgonów oraz tendencje zmian i dystans, jaki dzieli Polskę od innych krajów.

**Cel:**

Celem opracowania jest przedstawienie aktualnych danych oraz tendencji zmian od 2000 roku w zakresie poziomu i przyczyn umieralności dzieci i młodzieży w wieku 1-19 lat w Polsce na tle statystyk wzorcowych krajów europejskich.

**Materiał i metody:**

Dane pochodzą z krajowej bazy demograficznej udostępnianej przez Główny Urząd Statystyczny oraz z aktualnej wersji międzynarodowej bazy Światowej Organizacji Zdrowia (WHO-MDB). Polskę, należącą stale do grupy Eur-B, porównano z połączoną grupą 27 krajów zaklasyfikowanych przez WHO do grupy o bardzo niskiej ogólnej umieralności (Eur-A). Oszacowano trend liniowy umieralności w latach 2000-2013, ogółem i według wybranych przyczyn. Przyczyny zgonu przedstawiono według głównych klas X Rewizji Międzynarodowej Statystycznej Klasyfikacji Chorób i Problemów Zdrowotnych (ICD-10). Za nadrzędne kategorie przyjęto przyczyny zewnętrzne oraz pozostałe.

**Wyniki:**

W 2015 roku zarejestrowano w Polsce 1474 zgony dzieci i młodzieży w wieku 1-19 lat, co oznacza 19,9 przypadków na 100 000 populacji, odpowiednio 25,4 dla chłopców i 14,2 dla dziewcząt. Zmiany poziomu umieralności dzieci i młodzieży według wieku mają charakter nieliniowy ( U-kształtny), a najniższy jej poziom występuje w wieku 5-9 lat.

Według danych z 2014 roku, 50,2% zgonów dzieci i młodzieży w wieku 1-19 lat nastąpiło z przyczyn zewnętrznych o charakterze przypadkowym lub zamierzonym. Odsetek ten zwiększa się od 18,4% w wieku 1-4 lat, do 68,6% w wieku 15-19 lat. Poza przyczynami zewnętrznymi, dominują zgony z powodu nowotworów złośliwych, wad wrodzonych, chorób układu nerwowego i chorób układu oddechowego. Ranking przyczyn zgonów i ich udział procentowy zmienia się w kolejnych grupach wieku i na przestrzeni lat. Po pierwsze, w młodszych grupach wieku większy udział mają wrodzone wady rozwojowe i choroby układu oddechowego, podczas gdy u młodzieży zgony coraz częściej następują z powodu chorób układu krążenia i stanów nieokreślonych. Po drugie, we wszystkich grupach wieku wyeliminowane zostały zgony z powodu chorób zakaźnych, a zwiększył się udział chorób rzadkich.

Nadumieralność polskiej populacji w wieku 1-19 lat zwiększyła się w badanym okresie z 21% w 2000 r. do 56% w 2013 r., głównie z powodu mniej korzystnych tendencji zmian współczynników zgonów młodzieży. Tempo spadku umieralności małych dzieci (1-4 lat) okazało się w Polsce nawet szybsze niż w krajach Eur-A, co dotyczy zarówno przyczyn zewnętrznych, jak i pozostałych. W grupie wieku 5-14 lat szybsze tempo zmian utrzymuje się jedynie w odniesieniu do przyczyn zewnętrznych. U młodzieży i młodych dorosłych zwiększył się w badanym okresie dystans między Polską a krajami Eur-A. Różnice w tempie zmian na niekorzyść Polski dotyczyły w tej grupie wieku obu nadrzędnych kategorii przyczyn, ale były większe w odniesieniu do przyczyn zewnętrznych. Częściowo można to tłumaczyć pojawieniem się w Polsce od 2008 roku wzrostowego trendu samobójstw, współwystępującego ze stabilizacją analogicznych wskaźników w krajach Eur-A.

**Wnioski:**

Umieralność populacji w wieku 1-19 lat systematycznie w Polsce zmniejsza się, ale nadal przekracza przeciętny poziom wzorcowych krajów europejskich, szczególnie w odniesieniu do młodzieży. Oceniając szansę dalszego obniżenia umieralności w Polsce do poziomu w Eur-A, należy zwracać uwagę na przyczyny uznawane za możliwe do uniknięcia. W dalszych opracowaniach należałoby skoncentrować się na zasygnalizowanych w tym opracowaniu trendach i porównaniach międzynarodowych w odniesieniu do szczegółowych rozpoznań, dyskutując możliwości prewencji. Perspektywa wprowadzenia klasyfikacji ICD-11, poprzez zmiany w systemie kodowania, stwarza szansę lepszej oceny zagrożenia populacji w wieku rozwojowym chorobami rzadkimi, które stanowią nowe wyzwanie dla zdrowia publicznego.

## Wstęp

Analizy umieralności stanowią ważny element oceny sytuacji zdrowotnej społeczeństwa. Umieralność niemowląt i przeciętne trwanie życia są ciągle traktowane jako kluczowe wskaźniki rozwoju ekonomicznego [1]. Poziom umieralności dzieci powyżej 1 roku życia i młodzieży jest dużo niższy niż w grupie niemowląt i w populacji dorosłych, co skłania do poszukiwania alternatywnych wskaźników związanych z chorobowością w wieku 1-19 lat, w tym z obciążeniem chorobami o częstym występowaniu, ale o mniejszej śmiertelności. U starszej młodzieży, definiując kluczowe mierniki zdrowia, zwraca się większą uwagę na częstość występowania czynników ryzyka chorób przewlekłych (otyłość, niewłaściwa dieta, używania substancji psychoaktywnych, niedostateczna aktywność fizyczna) oraz problemy swoiste dla tej grupy wieku (zaburzenia zdrowia psychicznego, ciąże nieletnich, choroby przenoszone drogą płciową, w tym HIV/ AIDS, urazy z powodu wypadków) [2, 3, 4]. Niemniej jednak nie wolno ignorować statystyk umieralności w żadnej grupie wieku, ponieważ poprzez systematyczne ich monitorowanie można zidentyfikować problemy pojawiające się w poszczególnych grupach wieku, oceniając różnice w strukturze przyczyn zgonów. W dłuższym okresie duże znaczenie ma ocena tendencji zmian i dystansu, jaki dzieli dany kraj od innych.

Według raportów Światowej Organizacji Zdrowia (WHO), poziom umieralności dzieci i młodzieży stale się obniża, jednak nadal występują znaczne różnice między krajami [5]. Kraje przodujące wyznaczają realny poziom, do którego powinny dążyć kraje teoretycznie posiadające zbliżone warunki rozwoju [6, 7]. Przyczyny różnic między krajami są złożone i trudno jest podać jedno proste rozwiązanie, jak można byłoby je zmniejszyć.

Okresowo powtarzane analizy umieralności dzieci i młodzieży [8, 9], jak również opracowania na temat metodologii tego typu badań [10], są ważnym uzupełnieniem opracowań dotyczących zgonów okołoporodowych i w pierwszym roku życia [11, 12] oraz raportów na temat sytuacji zdrowotnej całej ludności Polski [13]. Inicjatywy okresowych analiz umieralności dzieci i młodzieży podejmują czasopisma pediatryczne i z obszaru zdrowia publicznego [14]. W statystykach globalnych większy nacisk kładzie się na kraje gorzej rozwinięte, co odwraca uwagę od problemów zdrowotnych starszych dzieci i młodzieży we względnie bogatszych krajach [4]. W 2014 roku czasopismo *Lancet* opublikowało serię artykułów [15, 16, 17] na temat przyczyn umieralności dzieci i młodzieży w krajach wysokorozwiniętych, wskazując na jej złożone uwarunkowania, w tym na współwystępowanie czynników biologicznych i psychologicznych wraz z wpływem środowiska fizycznego i społecznego, a także na rolę dostępności i jakości podstawowej i specjalistycznej opieki medycznej [18].

Dane dotyczące zdrowia populacji, w tym statystyki zgonów, są wykorzystywane dla usprawnienia działania systemu ochrony zdrowia na poziomie ogólnokrajowym i lokalnym. Wspomaga to politykę zdrowotną i programy zdrowotne na różnych etapach ich tworzenia, od ustalania grup ryzyka i priorytetów do oceny stopnia realizacji założonych celów [4]. Od ponad 30 lat w analizach umieralności, w ramach oceny jakości opieki zdrowotnej, zwraca się uwagę na zgony przedwczesne, czyli ludzi stosunkowo młodych oraz zgony z powodu chorób, które w świetle dostępnych technologii powinny być uleczalne (lub wręcz możliwe do uniknięcia przy odpowiedniej prewencji pierwotnej). W 1976 roku Rutstein i wsp. [19] opublikowali pierwszą listę około osiemdziesięciu takich chorób, która ostatnio została zmodyfikowana i zredukowana do 34 pozycji przez Nolte i McKee [20]. Klasy) kacje tego typu głównie odnoszą się do możliwości zapobiegania skutkom chorób ostrych i przewlekłych, podczas gdy wśród przyczyn zewnętrznych podkreśla się w tych zestawieniach konieczność ograniczania niepożądanych skutków zabiegów medycznych, szczególnie chirurgicznych.

W grupie dzieci i młodzieży występuje specyficzna struktura przyczyn zgonów, związana z dużo większym niż u osób dorosłych udziałem tzw. przyczyn zewnętrznych, które mogą wynikać ze zdarzeń przypadkowych lub działań zamierzonych.

Wymagają one innego podejścia do prewencji niż pozostałe choroby spowodowane przyczynami wewnętrznymi, obejmujące narządy i układy organizmu. Stanowią one największe obciążenie populacji w związku z potencjalnie utraconymi latami życia i latami życia z niepełnosprawnością. Obejmują one zarówno zgony z powodu wypadków komunikacyjnych, jak również w wyniku przemocy, w tym samobójstwa i zabójstwa. Powodzenie prewencji wypadkowej w dużej mierze zależy od czynników zlokalizowanych poza sektorem ochrony zdrowia, szczególnie w zakresie prewencji pierwotnej. Teoretycznie, wypadki (i ich skutki) w większości uznaje się za możliwe do uniknięcia, przy wdrożeniu skutecznych działań, szczególnie w obszarze legislacji i zmian środowiska ) zycznego. Zmiany te ukierunkowane są na poprawę bezpieczeństwa dzieci w środowisku wychowania, nauki, sportu i rekreacji, w tym poprawę bezpieczeństwa uczestników ruchu drogowego [21, 22].

W ostatnich latach coraz częściej podejmowany jest też temat chorób rzadkich lub ultrarzadkich w kontekście umieralności populacji w wieku rozwojowym, jak również w planowaniu kompleksowej opieki zdrowotnej i jej kosztów w odniesieniu do tej grupy pacjentów. Podnoszona jest również zasadność osobnej kodyfikacji tych chorób. Wiele chorób przewlekłych, w tym te rzadkie, zaczyna być bardziej eksponowana w statystykach stanu zdrowia starszych dzieci i młodzieży, z powodu ograniczenia śmiertelności w wieku niemowlęcym oraz wyeliminowania innych, dominujących wcześniej przyczyn. Zgodnie z definicją, choroby rzadkie to choroby zagrażające życiu lub powodujące przewlekłą niepełnosprawność, o maksymalnym regionalnym zasięgu od 1 na 10 000 do 1 na 1000 osób. W definicji Unii Europejskiej, zawartej w regulacji Parlamentu Europejskiego i Rady Europy z dnia 16 grudnia 1999 roku [23] podane jest kryterium częstości ich występowania nie większej niż 1 na 2000 osób. Natomiast choroby występujące z częstością 1 na 50 tysięcy lub mniejszą, to choroby ultrarzadkie. Aż 80 proc. chorób rzadkich i ultrarzadkich to choroby genetycznie uwarunkowane. Obecnie tylko niewielka liczba chorób rzadkich posiada międzynarodowe kody w systemach informacyjnych ochrony zdrowia, co uniemożliwia rutynowe analizy epidemiologiczne. Ich kody zostały dopasowane do dominującego obrazu klinicznego oraz zgodnie z bezpośrednią przyczyną zgonu. Etiologia ma zaś ciągle znaczenie wtórne, co powoduje że kody tych chorób są „rozsiane” w klasyfikacji ICD-10 w różnych grupach przyczyn lub wręcz nie ma odpowiednich rozpoznań w klasyfikacji.

Wydaje się, że aby analizy umieralności skutecznie wspomagały ocenę systemu opieki medycznej i stopnia wdrażania działań prewencyjnych, należy też odnieść się krytycznie do jakości uzyskiwanych danych. W świetle dostępnej wiedzy, mało jest tego typu analiz i częściej dotyczą one dorosłych niż dzieci i młodzieży [24]. Specjaliści z Głównego Urzędu Statystycznego [25] podkreślają, że statystyka publiczna ma ograniczony wpływ na prawidłowość wypełniania karty statystycznej przez lekarzy stwierdzających zgon (w części dotyczącej przyczyn), stanowiącej podstawę dla opracowania informacji na temat przyczyn zgonów. Występuje częste zjawisko wpisywania nazw przyczyn niezgodnie z obowiązującą klasyfikacją i wymaganym porządkiem, co skutkuje niską jakością danych statystycznych i w konsekwencji utrudnia znacząco ocenę sytuacji epidemiologicznej i zdrowotnej ludności Polski. Zwracają również uwagę na problem tzw. kodów śmieciowych (*garbage codes –* termin przyjęty przez WHO), będących skutkiem nieprawidłowych zapisów w karcie zgonu, uniemożliwiających nadanie odpowiedniego kodu przyczyny. Wydaje się także, że wprowadzenie przez Narodowy Fundusz Zdrowia w 2008 roku tzw. jednorodnych grup pacjentów (JGP) [26], mogło spowodować dodatkowe utrudnienia również w interpretacji kodowania przyczyn zgonów.

## Cel pracy

Celem opracowania jest przedstawienie aktualnych danych na temat umieralności dzieci i młodzieży w wieku 1-19 lat w Polsce oraz próba oceny jakości dostępnych informacji.

Sformułowano następujące pytania badawcze:

- Jaki jest obecny poziom umieralności dzieci i młodzieży w Polsce według płci i wieku?
- Jak kształtuje się umieralność dzieci i młodzieży w Polsce na tle wzorcowych krajów europejskich w świetle najnowszych danych?
- Czym różnią się obserwowane w Polsce trendy umieralności w grupach wieku oraz z powodu głównych kategorii przyczyn od trendów w innych krajach?
- Czy system kodowania przyczyn zgonów jest wystarczająco dokładny, aby ułatwić definiowanie obszarów priorytetowych w systemie opieki zdrowotnej nad dziećmi i młodzieżą.

## Materiał i metody

Wykorzystano ogólnie dostępne bazy danych demograficznych zamieszczone na stronie internetowej Głównego Urzędu Statystycznego (GUS). Szczegółowe dane dotyczące stanu ludności i zgonów według wieku znajdują się w zakładce „wyniki badań bieżących” (http://demografia.stat.gov.pl/bazademografia/Tables.aspx).

W zestawieniach międzynarodowych wykorzystano bazę WHO-MDB (*European Mortality Database*) dostępną na stronie internetowej Światowej Organizacji Zdrowia, wersja z lipca 2016 roku. Pozwala ona na interaktywne tworzenie zestawień w różnych przekrojach (http://www.euro.who.int/en/data-and-evidence/databases/european-health-for-all-database-hfa-db) Ograniczeniem tej bazy jest posługiwanie się z góry zdefiniowaną listą przyczyn zgonów oraz niejednolite zasady agregacji grup wieku w odniesieniu do różnych przyczyn. W związku z tym trudno było uzyskać wszystkie zaplanowane zestawienia, dokładnie dla przedziału wieku 1-19 lat.

Dane dotyczące Polski porównano z grupą 27 krajów zaliczanych do mających bardzo niski poziom umieralności dorosłych i dzieci (Eur-A) [27]. Są to: Andora, Austria, Belgia, Chorwacja, Cypr, Czechy, Dania, Finlandia, Francja, Grecja, Hiszpania, Holandia, Irlandia, Islandia, Izrael, Luksemburg, Malta, Monako, Niemcy, Norwegia, Portugalia, San Marino, Słowenia, Szwajcaria, Szwecja, Wielka Brytania i Włochy. Nadumieralność populacji polskiej w stosunku do krajów Eur-A liczono, jako różnicę standaryzowanych współczynników zgonów, wyrażoną procentowo w stosunku do współczynników w krajach Eur-A.

Przedstawiono dane z ostatniego dostępnego roku, co w przypadku krajowych zestawień opierających się na danych GUS oznacza 2015 rok lub 2014 rok (wg przyczyn), zaś 2013 rok w przypadku zestawień międzynarodowych uzyskanych z bazy WHO-MDB.

Współczynniki umieralności liczono na 100 000 osób danej grupy wieku, przyjmując za mianownik stan ludności na 30 czerwca danego roku. Niewielkie różnice w wartościach współczynników obliczanych na podstawie danych GUS względem zestawień WHO mogą wynikać ze standaryzacji oraz zdefiniowanej populacji bazowej. W porównaniach międzynarodowych zestawianych według wieku uwzględniono trzy dostępne grupy (1-4 lat, 5-14 lat i 15-24 lat), włączając też w niektórych analizach młodych dorosłych do 29 lat lub sporadycznie niemowlęta (jako łączna grupa 0-14 lat).

Przyczyny zgonu przedstawiono według głównych klas odpowiadających X Rewizji Międzynarodowej Statystycznej klasyfikacji Chorób i Problemów Zdrowotnych, klasyfikacja trzycyfrowa (z rozszerzeniem). Za nadrzędne kategorie przyjęto przyczyny zewnętrzne (klasy od V do Y) oraz pozostałe przyczyny (klasy od A do R). Lista klas przyczyn znajduje się dalej w tabeli II, natomiast ogólna klasyfikacja przyczyn zewnętrznych obejmuję: wypadki komunikacyjne, inne zewnętrzne przyczyny urazu wypadkowego (np. upadki, utonięcia, oparzenia, porażenia prądem), wypadki zamierzone (zabójstwa, samobójstwa) oraz powikłania opieki medycznej i chirurgicznej.

Źródłem danych o zgonach ludności Polski jest, dokument ,,Karta zgonu”, który zawiera informację o przyczynie zgonu bezpośredniej, wtórnej i wyjściowej. Przy opracowywaniu danych o zgonach według przyczyn przyjmuje się wyjściową przyczynę, czyli chorobę stanowiącą początek procesu chorobowego, który doprowadził do zgonu albo uraz czy zatrucie, w wyniku którego nastąpił zgon [28].

Analizując tendencję zmian, oszacowano trend liniowy w latach 2000-2013. Przyjęto umownie, że współczynnik regresji liniowej opisuje średnie tempo zmian umieralności w badanym okresie, a wyższa wartość współczynnika determinacji R-kwadrat (jakości dopasowania modelu) świadczy o lepszym dopasowaniu funkcji trendu do danych rzeczywistych.

## Wyniki i ich omówienie

### Obecny ogólny poziom umieralności

Według danych z 2015 roku, populacja dzieci i młodzieży w wieku 1-19 lat liczyła w Polsce 7,4 miliona osób. W porównaniu z początkiem okresu objętego analizą zmniejszyła się ona o około 2,3 mln osób, a w strukturze według wieku obniżył się udział starszej młodzieży, z chwilą gdy wiek adolescencji osiągnęły roczniki „niżu demogra) cznego”. Za ważną, ale trudną do wymiernego wykazania zmianę należy uznać wzrost udziału dzieci i młodzieży z przewlekłymi problemami zdrowotnymi, wynikający z coraz lepszej przeżywalności noworodków z mała masą urodzeniową i coraz lepszych możliwości leczenia chorób uznawanych wcześniej za letalne.

W 2015 roku zarejestrowano w Polsce 1474 zgony osób w wieku 1-19 lat, w tym 963 zgony chłopców (65%) i 511 dziewcząt (35%). Aktualne wskaźniki umieralności według wieku, płci i miejsca zamieszkania przedstawiono w tabeli I. Ogólny współczynnik umieralności wyniósł w tej grupie wieku 19,9 na 100 000 populacji, w tym 25,4 dla chłopców i 14,2 dla dziewcząt.

**Tabela I:** Umieralność dzieci i młodzieży według płci i wieku w 2015 r. Table I. Mortality in children and adolescents by age and gender in 2015.

Wiek (lata) *Age (yrs)*	Populacja *Population*	Zgony na 100 000 *Deaths per 100 000*		
		Ogółem *Total*	Chłopcy *Boys*	Dziewczęta *Girls*
1-19	7404081	19,9	25,4	14,2
1 - 4	1549126	17,0	18,6	15,3
1	371513	30,7	33,0	28,2
2	379144	15,8	16,9	14,7
3	392855	12,0	14,8	8,9
4	405614	10,4	10,6	10,2
5 - 9	2044035	9,3	9,5	9,1
10 - 14	1802451	13,5	15,9	10,9
15 - 19	2008469	38,7	55,2	21,3

Źródło: Opracowanie własne na podstawie danych GUS

Analizując umieralność dzieci i młodzieży w tradycyjnych grupach wieku, stwierdzono najniższy jej poziom w wieku 5-9 lat, a najwyższy w wieku 15-19 lat. Znaczący spadek umieralności można zauważyć w kolejnych rocznikach najmłodszej grupy wieku (1-4 lat). W drugim roku życia (12-24 miesiące) zanotowano 30,7 zgonów na 100 000 populacji, podczas gdy w piątym roku życia (48-60 miesięcy) już trzy razy mniej (10,4 na 100 000).

Na rycinie 1 przedstawiono współczynniki umieralności na 100 000 populacji według płci i dokładnych roczników od pierwszego do dziewiętnastego ukończonego roku życia. Różnice między chłopcami i dziewczętami zaczynają narastać w drugiej dekadzie życia, a po 14 roku życia są coraz większe. U dziewcząt zagrożenie zgonem jest zbliżone w skrajnych rocznikach (krzywa ma dokładny kształt litery U), podczas gdy u chłopców różnica między skrajnymi rocznikami jest trzykrotna, na niekorzyść młodzieży.

**Ryc. 1:**
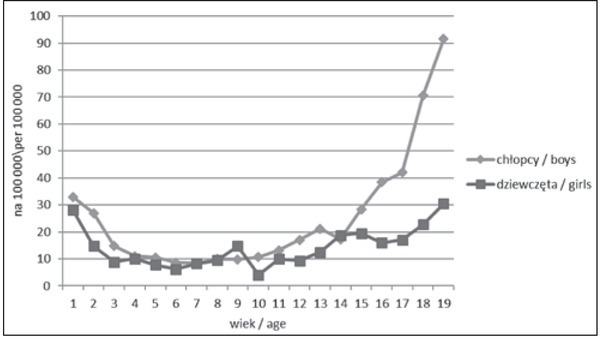
Zgony dzieci i młodzieży w 2015 roku na 100 000 osób według płci i dokładnego wieku (dane: GUS). Fig. 1. Deaths in children and adolescents in 2015 per 100 000 persons by gender and exact age (data:GUS).

Na podobne prawidłowości wskazywały poprzednie opracowania danych polskich, jak również zestawienia zagraniczne [8, 9, 14]. Zwraca się uwagę na drugi i trzeci roku życia, kiedy dają o sobie znać problemy zdrowotne podobne jak u niemowląt w okresie po-noworodkowym, wynikające ze skróconego czasu trwania ciąży lub wrodzonych wad rozwojowych. W porównaniu z okresem od 2 do 12 miesiąca życia, w drugim roku życia zgony są już ponad 3 razy rzadsze. Drugim okresem krytycznym jest wiek adolescencji. O ile, jeszcze 30 lat temu umieralność małych dzieci przewyższała poziom obserwowany w wieku 15-19 lat [8], o tyle obecnie ryzyko zgonu nastolatka jest dużo większe. Jak wykazane zostanie dalej, przyczyny zgonów starszej młodzieży są bardziej zróżnicowane, gorzej diagnozowane, a kierunki działań zapobiegawczych są bardziej rozproszone.

### Główne klasy przyczyn zgonów dzieci i młodzieży

W tabeli II zestawiono przyczyny zgonów dzieci i młodzieży według danych z 2014 roku, podając liczby bezwzględne zarejestrowanych przypadków oraz częstość zgonów na 100 000 populacji. Biorąc pod uwagę klasy ICD-10, stwierdzono, że 98,6% zgonów dotyczy dziewięciu głównych klas, podczas gdy 1,4% zgonów, klas pozostałych (włączając nowotwory niezłośliwe będące w klasie D).

**Tabela II:** Zgony dzieci i młodzieży według klas przyczyn zgonu i wieku w 2014 roku. Table II. Deaths in children and adolescents by classes of causes and age in 2014.

Przyczyny *Causes*	1-19 lat *1-19 yrs*	1-4 lat *1-4 yrs*	w tym / *in which*:				5-9 lat *5-9 yrs*	10-14 lat *10-14 yrs*	15-19 lat *15-19 yrs*
			1	2	3	4			
Liczba zgonów / *Number of deaths*									
AB	14	8	4	2	1	1	2	0	4
CD	215	56	17	11	17	11	43	48	68
E	38	9	1	5	2	1	7	7	15
G	104	21	5	6	6	4	23	25	35
I	66	8	5	3	0	0	3	9	46
J	92	30	13	7	5	5	23	14	25
Q	154	86	51	14	13	8	28	19	21
R	57	4	2	2	0	0	8	4	41
VWXY	769	51	11	11	16	13	50	96	572
Inne* Others*	22	4	1	2	0	1	5	6	7
Ogółem *Total*	1531	277	110	63	60	44	192	228	834
na 100 000 osób / *per 100 000*									
AB	0,2	0,5	1,1	0,5	0,2	0,2	0,1	0,0	0,2
CD	2,9	3,6	4,5	2,8	4,2	2,6	2,2	2,6	3,3
E	0,5	0,6	0,3	1,3	0,5	0,2	0,4	0,4	0,7
G	1,4	1,3	1,3	1,5	1,5	0,9	1,2	1,4	1,7
I	0,9	0,5	1,3	0,8	0,0	0,0	0,2	0,5	2,2
J	1,2	1,9	3,4	1,8	1,2	1,2	1,2	0,8	1,2
Q	2,1	5,5	13,5	3,6	3,2	1,9	1,4	1,0	1,0
R	0,8	0,3	0,5	0,5	0,0	0,0	0,4	0,2	2,0
VWXY	10,3	3,2	2,9	2,8	3,9	3,1	2,5	5,3	27,5
Inne* Others*	0,3	0,3	0,3	0,5	0,0	0,2	0,3	0,3	0,3
Ogółem *Total*	20,5	17,6	29,0	16,0	14,8	10,4	9,7	12,5	40,1
AB -	Wybrane choroby zakaźne i pasożytnicze *Certain infectious and parasitic diseases*				Q -	Wady rozwojowe wrodzone, zniekształcenia i aberracje chromosomowe *Congenital malformations, deformations and chromosomal abnormalities*			
CD -	Nowotwory złośliwe i *in situ Malignant neoplasms and in situ*				R -	Objawy, cechy chorobowe oraz nieprawidłowe wyniki badań klinicznych i laboratoryjnych niesklasyfikowane gdzie indziej *Symptoms, signs and abnormal clinical and laboratory findings, not elsewhere classified*			
E -	Zaburzenia wydzielania wewnętrznego, stanu odżywienia i przemian metabolicznych *Diseases of the blood and blood-forming organs and certain disorders involving the immune mechanism*				V do Y	Urazy, zatrucia i inne określone skutki działania czynników zewnętrznych *External causes of morbidity and mortality*			
G -	Choroby układu nerwowego *Diseases of the nervous system*					Pojedyncze przypadki w klasie D50+, F, K, N, O i P			
I -	Choroby układu krążenia *Diseases of the circulatory system*				*Inne * Others	Zero przypadków w klasie H,L i M *Single cases in the class D50+, F, K, N, O i P*			
J -	Choroby układu oddechowego *Diseases of the respiratory system*					*None case in the class H,L i M*			

Źródło: Opracowanie własne na podstawie danych GUS

Połowa zgonów nastąpiła z tzw. **przyczyn zewnętrznych** – niezamierzonych, zamierzonych lub o nieustalonej intencji (klasa V-Y). W 2014 roku zmarło z tej przyczyny 769 osób w wieku 1-19 lat. Udział przyczyn zewnętrznych w statystyce zgonów systematycznie zwiększa się w kolejnych grupach wieku − od 18,4% w wieku 1-4 lat, do 68,6% w wieku 15-19 lat.

Drugą klasę przyczyn zgonów stanowią **nowotwory**. Kategoria ta obejmuje głównie nowotwory złośliwe (klasa C). W 2014 roku zmarło z tej przyczyny 214 osób w wieku 1-19 lat, co stanowi 14,0% wszystkich zgonów. Współczynniki na 100 000 populacji są nieznacznie podwyższone w skrajnych grupach wieku w porównaniu z dziećmi w wieku 5-14 lat.

Trzecią z kolei klasę przyczyn zgonów dzieci i młodzieży stanowią **wrodzone wady rozwojowe** (klasa Q). W 2014 roku zmarły z tej przyczyny 154 osoby w wieku 1-19 lat, co stanowi 10,1% wszystkich zgonów. Współczynniki zgonów na 100 000 populacji wyraźnie obniżają się w kolejnych grupach wieku. W drugim i trzecim roku życia jest to jeszcze dominująca przyczyna, przewyższająca udział wypadków i nowotworów złośliwych. W opracowaniach dotyczących całej populacji (łącznie z dorosłymi) wady wrodzone są niezauważaną przyczyną zgonów. Według danych z 2014 roku, 77% zgonów z powodu wrodzonych wad rozwojowych nastąpiło w pierwszym roku życia, 8% w wieku 1-19 lat i jedynie 15% u osób dorosłych, od 20 roku życia.

Kolejną ważną klasą przyczyn zgonów dzieci i młodzieży są **choroby układu nerwowego** (klasa G). W 2014 roku zmarły z tej przyczyny 104 osoby w wieku 1-19 lat, co stanowi 6,8% wszystkich zgonów. Udział procentowy tej klasy chorób w rankingu przyczyn umieralności zmienia się w kolejnych grupach wieku, choć wartości współczynników utrzymują się na stabilnym poziomie. Wobec ogólnie niskiej umieralności, w wieku 10-14 lat, choroby układu nerwowego zajmują obecnie trzecią pozycję w rankingu przyczyn zgonów.

Spośród pozostałych klas przyczyn zgonów dzieci i młodzieży, warto zwrócić uwagę na **choroby układu oddechowego** (klasa J − 92 przypadki w 2014 roku), **choroby układu krążenia** (klasa I − 66 przypadków) oraz **objawy i stany niezaklasyfikowane wcześniej** (klasa R − 57 przypadków). Choroby układu oddechowego stanowią większe zagrożenie dla młodszych dzieci, podczas gdy współczynniki zgonów z powodu chorób układu krążenia oraz stanów niedokładnie zaklasyfikowanych zwiększają się u młodzieży w wieku 15-19 lat.

Wyróżnienie w tabeli II **chorób zakaźnych i pasożytniczych** (połączona klasa A i B) jako jednej z dziewięciu najważniejszych klas przyczyn zgonów zaczyna mieć w świetle najnowszych statystyk znaczenie historyczne. W 2014 roku zanotowano w wieku 1-19 lat tylko 14 przypadków zgonów z tej przyczyny. Stosunkowo częściej notowane są one do 36 miesiąca życia. Można więc byłoby zacząć włączać tę klasę do „innych przyczyn”.

### Szczegółowa analiza przyczyn zgonów dzieci i młodzieży

Analizując przyczyny zgonów według szczegółowych kodów ICD-10, można zauważyć znaczną ich różnorodność oraz poważne problemy klasyfikacyjne. W 2014 roku 1531 zgonów dzieci i młodzieży w wieku 1-19 lat zostało zakodowanych według 415 różnych przyczyn (kod trzycyfrowy z rozszerzeniem), z czego w 247 przypadkach był to pojedynczy przypadek. Zidentyfikowano 521 zgonów zakodowanych z rozszerzeniem „9”, co oznacza przypadek bliżej nieokreślony (BNO). Tego typu tendencja do wybierania rozpoznań BNO występowała już wcześniej. Na przykład, dziesięć lat wcześniej, w 2005 roku udział tego typu rozpoznań wynosił w tej samej grupie wieku nawet 43%.

Wśród chorób zakaźnych i pasożytniczych, dominują zgony z powodu posocznicy. Należy pamiętać, że są to pojedyncze przypadki (A39-A41 − 9 zgonów w wieku 1-19 lat).

W klasie nowotworów największa liczba zgonów dotyczyła nowotworów złośliwych mózgu (C71 − 87 przypadków) i ostrej białaczki limfoblastycznej (C91.0 – 24 przypadki). W odniesieniu do pozostałych rozpoznań z klasy C zanotowano w 2014 roku pojedyncze przypadki zgonów dzieci i młodzieży wieku 1-19 lat, w tym najwięcej z powodu: ziarnicy i chłoniaków (12 zgonów) nowotworów złośliwych kości (10 zgonów), nowotworów złośliwych tkanek miękkich (9 zgonów) oraz nowotworów złośliwych nerki (6 zgonów). Bez retrospektywnego wywiadu trudno jest ocenić, jak często u tej samej osoby pojawiły się tzw. drugie pierwotne nowotwory.

W klasie „E” zaburzeń wydzielania wewnętrznego, stanu odżywienia i przemian metabolicznych, w wieku 1-19 lat dominowały jako przyczyna zgonów choroby metaboliczne, np. zwłóknienie wielotorbielowate (różne postacie mukowiscydozy E84 – 10 przypadków), rzadkie choroby pod postacią zaburzeń spichrzania lipidów (E75 – 9 przypadków) lub mukopolisacharydozy (E76.0 – 6 przypadków). Z powodu różnych powikłań cukrzycy insulinozależnej (E10.7 i E10.8) zmarły 3 osoby. Pozostałe zgony w tej klasie dotyczyły pojedynczych przypadków spowodowanych innymi rzadkimi chorobami metabolicznymi. Warto podkreślić, że na tle ogólnych zestawień, klasa „E” kojarzona jest głównie z cukrzycą i jej powikłaniami (E10-E14), co potwierdza obserwacja, że w 2014 roku 94% zgonów całej ludności kodowanych w klasie „E” nastąpiło z tej przyczyny.

W klasie chorób układu nerwowego najwięcej przypadków zgonów spowodowanych było dziecięcym porażeniem mózgowym (G80 – 36 przypadków, w tym 30 przypadków kodowanych jako G80.9, czyli mózgowe porażenie dziecięce nieokreślone), padaczką (G40 – 19 przypadków, z czego 17 kodowane jako padaczka nieokreślona G40.9). Tak dominująca część BNO w tej grupie spowodowana była być może podaniem kodu choroby według głównej manifestacji klinicznej, a zmarłe dzieci mogły mieć inne zaburzenia, najczęściej genetyczne, będące przyczyną między innymi padaczki. Kolejnymi przyczynami zgonów były pierwotne zaburzenia mięśniowe z subklasy chorób połączeń nerwowo-mięśniowych (G71 – 15 przypadków) oraz zapalenie mózgu i/lub rdzenia kręgowego (G04 – 12 przypadków, też głównie kodowane jako nieokreślone zapalenia mózgu i/lub rdzenia kręgowego G04.9).

W klasie chorób układu krążenia, zgony najczęściej następowały z powodu zatrzymania akcji serca (I46 – 22 przypadki), rzadziej z powodu niewydolności serca (I50 - 8 przypadków). Dominowały zgony młodzieży w wieku 15-19, a połowa przypadków była również niedokładnie określona (I46.9 i I50.9).

W klasie chorób układu oddechowego w 2014 roku zanotowano najwięcej zgonów z powodu zapalenia płuc (J12 do J18 − łącznie 76 przypadków), w tym szczególnie zapalenia płuc wywołanego przez niezidentyfikowany czynnik zakaźny (J18 – 58 przypadków).

W statystyce zgonów w wieku 1-19 z powodu wrodzonych wad rozwojowych dominowały wady układu krążenia (Q20-Q28: łącznie 84 przypadki), w tym najczęściej „inne wrodzone wady rozwojowe serca” (Q24 – 31 przypadków, w tym 20 przypadków kodowanych jako Q24.9 czyli, wrodzone wady serca rozwojowe nieokreślone). Na dalszym miejscu według częstości zgonów były wrodzone wady rozwojowe układu nerwowego (Q00 – Q07 − 20 przypadków, głównie wodogłowie − 9) oraz aberracje chromosomowe niesklasyfikowane gdzie indziej (Q89 – Q99 − 14 przypadków, w tym 7 - zespół Edwardsa i 1 – zespół Pataua).

W klasie „R” objawów, cech chorobowych i nieprawidłowych wyników badań niesklasyfikowanych gdzie indziej, przyczyną zgonu prawie w stu procentach były „niedokładnie określone lub inne przyczyny zgonu”, kodowane jako R96 do R99. Na 56 takich przypadków zarejestrowanych w 2014 roku, 41 dotyczyło młodzieży w wieku15-19 lat. Opracowania zagraniczne wskazują na znaczne zróżnicowanie między krajami, jeśli chodzi o dopuszczanie klasyfikacji przyczyn zgonów do klasy R. Wykazano na przykład powszechne stosowanie tego typu praktyki w Danii, wobec 13-krotnie niższych odpowiednich współczynników w Finlandii [29].

Wśród nieintencjonalnych zewnętrznych przyczyn zgonów dzieci i młodzieży w wieku 1-19 lat zarejestrowano w 2014 roku 314 zgony w wypadkach komunikacyjnych (V1-V99). Przeważnie były to wypadki użytkownika samochodu (124 zgony w klasie V40-V49) lub osoby pieszej (68 zgonów w klasie V3-V9). Poza wypadkami komunikacyjnymi, występuje kategoria tzw. urazów wypadkowych (W00-X59), gdzie zanotowano przede wszystkim śmiertelne utonięcia (49 przypadków, W67-W74) oraz wypadkowe zatrucia (22 przypadki − X41-X49).

Wypadki spowodowane działaniami zamierzonymi obejmują zamierzone samouszkodzenie oraz napaść, co w przypadku zdarzeń ze skutkiem śmiertelnym oznacza odpowiednio samobójstwo lub zabójstwo. W 2014 roku w populacji dzieci i młodzieży zarejestrowano 208 przypadków samobójstw (X60-X84), w tym 13 w wieku 10-14 lat i 195 w wieku 15-19 lat. Osoby w wieku 1-19 lat rzadko były o) arami zabójstwa (15 przypadków w 2014 r. wg kodów X85-Y09). Liczba zgonów spowodowanych zdarzeniami o nieokreślonym zamiarze była większa (Y11-Y34 – 53 przypadki).

Podsumowując analizę szczegółowych przyczyn zgonów, warto zwrócić uwagę na choroby, które w świetle standardów międzynarodowych nie powinny stanowić przyczyny zgonu (najczęściej uwzględnia się przypadki zgonu przed 75 rokiem życia) przy odpowiedniej opiece medycznej. Są to głównie przyczyny pozawypadkowe. Posługując się listą kodów ICD-10 i aktualnie obowiązującą na świecie listą chorób uleczalnych i do uniknięcia [20], stwierdzono w 2014 roku w grupie wieku 1-19 lat 270 zgonów, w tym tylko 3 w klasie przyczyn zewnętrznych (powikłania zabiegów medycznych). Oznacza to, że jedną trzecią zgonów osób w wieku 1-19 lat, które nastąpiły z przyczyn innych niż zewnętrzne należy uznać za możliwe do uniknięcia. Znacząca liczba przypadków dotyczyła zapaleń płuc, wad wrodzonych serca oraz padaczek (poza padaczkami BNO, w przypadku których zgon mógł nastąpić w wyniku nierozpoznanych, nieuleczalnych chorób zwyrodnieniowych mózgu lub rzadkich chorób metabolicznych o podłożu genetycznym).

### Tendencje zmian umieralności dzieci i młodzieży na tle danych międzynarodowych

W tabeli III przedstawiono trendy umieralności dzieci i młodzieży w wieku 1-19 lat w latach 2000-2013, porównując Polskę z 27 wzorcowymi krajami, to znaczy krajami o najniższej umieralności (Eur-A). W całej grupie 1-19 lat, zarówno w Polsce, jak i w krajach Eur-A zanotowano wyraźny spadek częstości zgonów. W Polsce występowały okresowe wahania z ogólną tendencją spadkową. W krajach Eur-A występowała natomiast jednolita tendencja spadkowa, a tempo spadku było szybsze niż w Polsce (odpowiednio średnio rocznie współczynnik zgonów obniżał się o 0,818 i o 0,587). W związku z wolniejszym tempem zmian ponad dwukrotnie zwiększyła się nadumieralność populacji polskiej. Wskaźnik nadumieralności wynosił 56,2% w 2013 roku, wobec 20,7% w 2000 roku. W stosunku do krajów przodujących wewnątrz grupy Eur-A dystans jest jeszcze większy. W 2013 roku w 7 krajach umieralność dzieci i młodzieży spadła do poziomu poniżej 13 na 100 000 ludności (Luksemburg 7,68; Malta – 9,10; Hiszpania – 12,48; Holandia – 12,63; Szwecja – 12,70; Norwegia – 12,84).

**Tabela III:** Trendy umieralności dzieci i młodzieży w wieku 1-19 lat na 100 000 osób, według dwóch głównych kategorii przyczyn. Table III. Trends in mortality of children and adolescents aged 1-19 per 100 000 persons by main two categories of causes.

	Ogółem *Total*		Zewnętrzne przyczyny *External causes*		Pozostałe przyczyny *Other causes*	
	Polska	Eur-A	Polska	Eur-A	Polska	Eur-A
2000	29,71	24,62	15,15	11,87	14,56	12,75
2001	29,34	24,25	14,65	11,44	14,69	12,81
2002	30,29	23,20	15,75	10,77	14,54	12,43
2003	27,01	22,27	14,46	10,12	12,55	12,15
2004	25,89	20,92	13,02	9,60	12,87	11,32
2005	26,80	20,16	14,09	8,87	12,71	11,29
2006	26,92	19,15	12,89	8,31	14,03	10,84
2007	26,91	18,83	13,98	8,17	12,93	10,66
2008	26,83	17,95	14,22	7,58	12,61	10,37
2009	25,82	17,47	12,85	7,11	12,97	10,36
2010	22,76	16,15	11,04	6,30	11,72	9,85
2011	23,04	15,67	11,35	6,03	11,69	9,64
2012	22,34	14,82	11,21	5,53	11,13	9,29
2013	22,40	14,34	11,03	5,27	11,37	9,07
zmiany *annual* roczne *rate*	-0,587	-0,818	-0,339	-0,520	-0,248	-0,298
R^2^	0,847	0,992	0,784	0,994	0,758	0,981

Źródło: Opracowanie własne na podstawie danych WHO-MDB R^2^ – współczynnik dopasowania linii regresji

W odniesieniu do przyczyn poza wypadkowych, średni roczny spadek współczynnika zgonów oszacowano na 0,248 w Polsce i 0,298 w krajach Eur-A. W odniesieniu do przyczyn zewnętrznych tempo zmian było wyraźnie wolniejsze w Polsce niż w krajach Eur-A (spadek o 0,339 i 0,520 na 100 000 osób). Jeśli ograniczymy analizy do tzw. zdarzeń niezamierzonych (wypadki, kody V01-X59), to umieralność obniżała się średnio rocznie o 0,324 w Polsce oraz o 0,450 zgonów na 100 000 osób w wieku 1-19 lat w krajach Eur-A.

Powyższe tendencje zmian przełożyły się na zmianę wskaźnika nadumieralności polskiej populacji dzieci i młodzieży. Podczas gdy w przypadku przyczyn zewnętrznych nadumieralność wzrosła w latach 2000-2013 z 27,6% aż do 109,3%, w odniesieniu do pozostałych przyczyn zgonów był do wzrost z 14,2% do 25,4%.

W tabeli IV podsumowano tendencje zmian umieralności dzieci i młodzieży z powodu przyczyn zewnętrznych i pozostałych przyczyn, uwzględniając trzy grupy wieku. Ilustracją tych zmian są ryciny 2 i 3. W zestawieniu z wcześniejszą analizą dotycząca całej grupy 1-19 lat, w młodszych grupach wieku zasadniczo zmienia się wnioskowanie na temat tempa zmian umieralności w Polsce na tle wzorcowych krajów europejskich. W wieku 1-4 lat jest ono w Polsce szybsze niż w krajach Eur-A, co dotyczy zarówno przyczyn zewnętrznych, jak i pozostałych. W grupie wieku 5-14 lat szybsze tempo zmian utrzymuje się jedynie w odniesieniu do przyczyn zewnętrznych. Uzyskanie korzystniejszych niż w krajach Europy Zachodniej trendów zgonów młodszych roczników można wiązać z poprawą jakości systemu ratownictwa w Polsce, szczególnie ratownictwa medycznego, m.in. poprzez stworzenie jednolitego systemu do obsługi zgłoszeń alarmowych, kierowanych do numerów alarmowych [29]. Inną ważnym czynnikiem wpływającym na redukcję zgonów dzieci są zmiany legislacyjne i skuteczniejsze egzekwowanie prawa, szczególnie w zakresie bezpieczeństwa drogowego dziecka [30].

**Tabela IV:** Średnie roczne zmiany współczynników umieralności dzieci i młodzieży według wieku i dwóch głównych kategorii przyczyn (2000-2013). Table IV. Average annual change in mortality in children and adolescents by age and main two categories of causes (2000-2013).

	1-4 lat *1-4 yrs*		5-14 lat *5-14 yrs*		15-24 lat *15-24 yrs*	
	Polska	Eur-A	Polska	Eur-A	Polska	Eur-A
Przyczyny zewnętrzne/*External causes*						
Zmiany roczne* Annual change*	-0,379	-0,296	-0,355	-0,228	-0,496	-1,473
R^2^	0,834	0,981	0,928	0,980	0,648	0,996
Pozostałe przyczyny/*Other causes*						
Zmiany roczne* Annual change*	-0,621	-0,448	-0,152	-0,191	-0,175	-0,445
R^2^	0,835	0,962	0,556	0,963	0,617	0,973

*Współczynniki regresji liniowej, istotne na poziomie p<0,001 R2 – współczynnik dopasowania linii regresji

**Ryc. 2:**
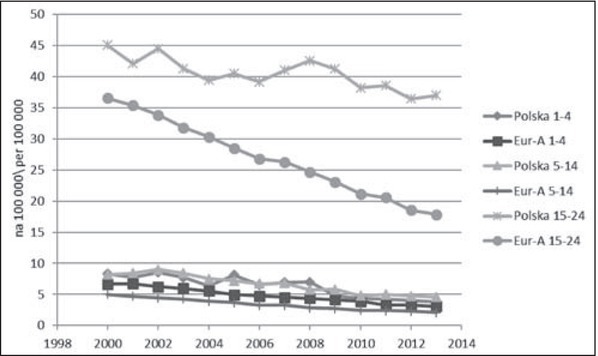
Umieralność z powodu przyczyn zewnętrznych w wieku 1-24 lat w Polsce i krajach Eur-A (dane: WHO-MDB). Fig. 2. Mortality from external causes in age 1-24 in Poland and Eur-A countries (data: WHO-MDB).

U młodzieży i młodych dorosłych różnice w tempie zmian na niekorzyść Polski dotyczyły obu głównych kategorii przyczyn, ale były większe w odniesieniu do przyczyn zewnętrznych (ryc. 3). Częściowo można to tłumaczyć pojawieniem się w Polsce od 2008 roku wzrostowego trendu samobójstw. Baza WHO-MDB dostarcza szczegółowych danych na temat samobójstw tylko dla grupy 15-29 (ryc. 4). Do roku 2007 występowała w Europie ogólna tendencja spadkowa, po czym w Polsce pojawił się trend wzrostowy, a w krajach Eur-A stabilizacja wskaźników. Dane z 2014 r. wskazują na utrzymanie się tendencji wzrostowej w Polsce i najwyższą od 2000 r. umieralność młodzieży i młodych dorosłych z powodu samobójstw (12,91 na 100 000). Interpretując te trendy, należy zwrócić uwagę, że w Polsce realizowany jest Narodowy Program Ochrony Zdrowia Psychicznego [31], w ramach którego minister właściwy do spraw oświaty i wychowania jest odpowiedzialny za realizację zadań w zakresie promocji zdrowia psychicznego i zapobiegania zaburzeniom psychicznym u dzieci i młodzieży szkolnej. Program realizowany w latach 2011-2015 został negatywnie oceniony przez Najwyższą Izbę Kontroli, która stwierdziła nieosiągnięcie założonych celów i niezrealizowanie większości zadań zaplanowanych, zarówno przez administrację rządową, jak i samorządową [32]. Oceniono, że nie doprowadzono do ograniczenia występowania zagrożeń dla zdrowia psychicznego, poprawy jakości życia osób z zaburzeniami psychicznymi i ich bliskich oraz lepszej dostępności świadczeń opieki zdrowotnej. W ramach Programu nie zrealizowano zadań związanych z redukcją rocznych wskaźników samobójstw dokonanych i prób samobójczych, a wręcz przeciwnie, w okresie realizacji Programu nastąpił wzrost liczby zamachów samobójczych zakończonych zgonem, również w grupie młodzieży.

**Ryc. 3:**
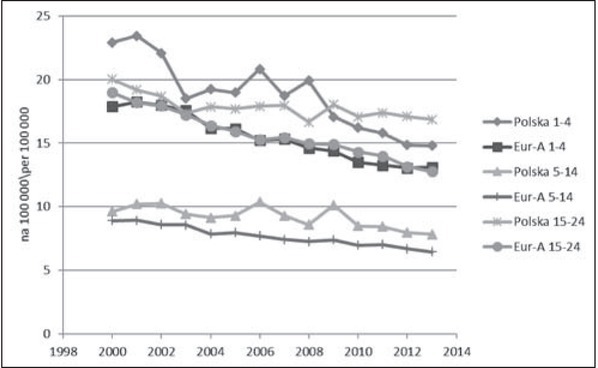
Umieralność z powodu przyczyn innych niż zewnętrzne w wieku 1-24 lat w Polsce i krajach EU-A (dane: WHO-MDB). Fig. 3. Mortality from other than external causes in age 1-24 yrs in Poland and Eur-A countries (data: WHO-MDB).

**Ryc. 4:**
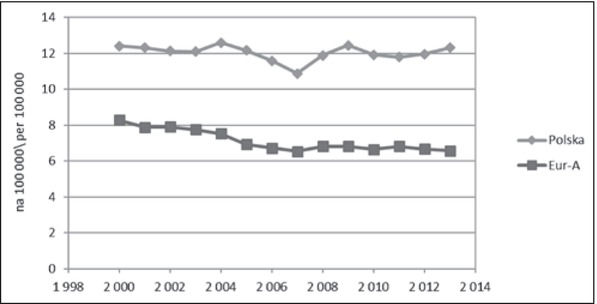
Umieralność z powodu samobójstw osób w wieku 15-29 lat w Polsce i krajach Eur-A (dane: WHO-MDB). Fig. 4. Suicide-related mortality in age 15-29 yrs in Poland and Eur-A countries (data: WHO-MDB).

Obszerniejsza analiza tendencji zmian umieralności z powodu szczegółowych przyczyn, w tym przyczyn uznanych za możliwe do uniknięcia wymaga odrębnego opracowania. Można jedynie wskazać kilka przykładów, które świadczą czasem na korzyść, a czasem na niekorzyść Polski. O jakości systemu kodowania przyczyn i pośrednio też o jakości opieki medycznej świadczy udział przyczyn nieokreślonych w statystyce zgonów (klasa R). Według zaleceń WHO, wpisanie kodu z klasy R w karcie zgonu jest ostatecznością, w przypadku gdy wyczerpano możliwości uzyskania precyzyjnej diagnozy. W porównaniu z krajami Eur-A, w Polsce notowana jest niższa umieralności z tych przyczyn u młodszych dzieci, ale wyższa u starszych dzieci i u młodzieży (ryc. 5).

**Ryc. 5:**
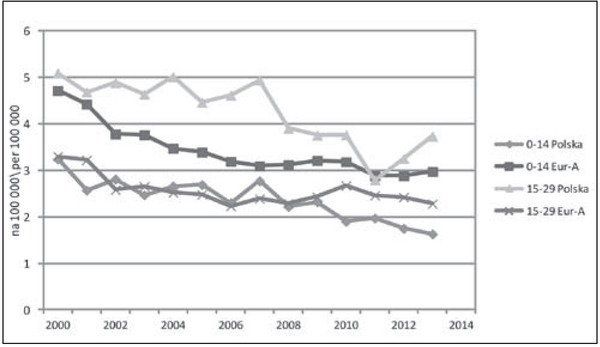
Umieralność niemowląt i dzieci oraz młodzieży i młodych dorosłych z powodu przyczyn kodowanych w klasie R w Polsce i krajach Eur-A (dane: WHO-MDB). Fig. 5. Mortality of infants & children and adolescents & young adults from causes clasified as class R in Poland and Eur-A countries (data: WHO-MDB).

U młodzieży i młodych dorosłych zarysował się do 2011 roku silny trend spadkowy, owocujący zrównaniem współczynników z krajami Eur-A, jednak tej korzystnej tendencji nie udało się utrzymać w latach 2012-14. Alarmujące z kolei wydają się zestawienia dotyczące zgonów z powodu zapaleń płuc, uznanych za typową przyczynę możliwą do uniknięcia (ryc. 6 − kody J12-J18). W świetle danych z 2013 roku, zapalenia płuc były w Polsce odpowiedzialne za 6,5% zgonów w wieku 1-19 lat, a od 2000 roku zarysowała się tendencja wzrostowa współczynników zgonów z tej przyczyny. W krajach Eur-A pojawiła się zaś wyraźna tendencja spadkowa, a udział zapaleń płuc w ogólnej liczbie zgonów wynosił w ostatnim roku tylko 1,2%.

**Ryc. 6:**
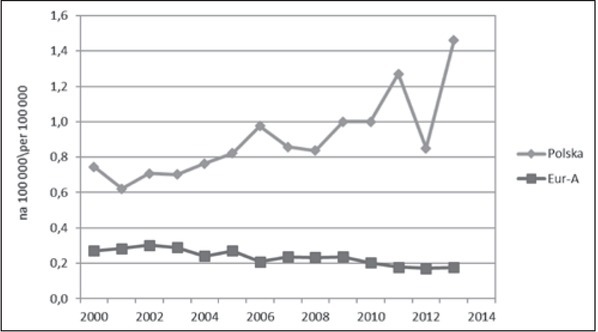
Umieralność dzieci i młodzieży w wieku 1-19 lat z powodu zapalenia płuc w Polsce i krajach Eur-A (dane: WHO-MDB). Fig. 6. Mortality of children and adolescents aged 10-19 yrs from pneumonia in Poland and Eur-A countries (data: WHO-MDB).

## Podsumowanie

W opracowaniu przedstawiono aktualne statystyki umieralności dzieci i młodzieży w Polsce na tle zestawień międzynarodowych. Obecnie liczba zgonów w populacji w wieku 1-19 lat oscyluje wokół 1500 przypadków (1531 w 2014 roku oraz 1474 w 2015 roku). Współczynniki zgonów dzieci i młodzieży są w Polsce nadal wyższe niż w przodujących krajach regionu europejskiego, ale dystans względem krajów wzorcowych stopniowo się redukuje. Za punkt odniesienia przyjęto 27 krajów Eur-A, o niższej umieralności dorosłych i dzieci. Grupa ta obejmuje większość krajów pierwotnej Unii Europejskiej (UE-15), Islandię, Izrael, Norwegię, Szwajcarię oraz wybrane tzw. małe państwa (Andora, Monako, San Marino). Pod tym względem opracowanie różni się od innych, uznających za punkt odniesienia całą Unię Europejską lub tzw. kraje UE-15 [13]. Polska jest ciągle w grupie Eur-B, jak również siedem innych krajów, które w tym samym czasie lub później wstąpiły do Unii (Bułgaria, Estonia, Litwa, Łotwa, Rumunia, Słowacja, Węgry). Należy jednak zwrócić uwagę, że na klasyfikację do poszczególnych grup (A, B, C) wpływa też poziom umieralności osób dorosłych.

W analizowanym okresie 2000-2014, zanotowano w Polsce wyraźną tendencję spadkową umieralności dzieci i młodzieży. Tempo spadku zmienia się jednak w grupach wieku i według przyczyn zgonów. Niewątpliwym sukcesem ostatnich lat jest bardzo szybki spadek umieralności młodszych dzieci (1-4) z powodu przyczyn zewnętrznych i pozostałych oraz starszych dzieci z powodu przyczyn zewnętrznych. Stosunkowo niski udział przyczyn nieokreślonych w statystyce zgonów małych dzieci też przemawia na korzyść systemu opieki zdrowotnej nad tą grupą wieku. Niepokoić powinno natomiast zbyt wolne tempo spadku umieralności młodzieży i młodych dorosłych oraz wysoki udział przyczyn nieokreślonych w statystyce zgonów. W całej populacji wieku rozwojowego należy zwracać też uwagę na okresy wzrostu częstości zgonów z wybranych przyczyn.

Prace wskazujące na młodzież jako grupę wieku zaniedbywaną w opiece medycznej pojawiają się od 30 lat. Zaowocowało to między innymi opracowaniem przez WHO globalnej strategii H4WA (*Health for the World’s Adolescents: a second chance in the second decade*) [4]. Doceniając w niej znaczenie monitorowania umieralności młodzieży, zwraca się uwagę na konieczność analizowania szeregu innych wskaźników związanych ze stylem życia w kontekście uwarunkowań społeczno-ekonomicznych. Według prezentowanego przez nas opracowania, prawie 70% zgonów w wieku 15-19 lat następuje z przyczyn zewnętrznych, które w większości też są możliwe do uniknięcia. Wśród przyczyn pozostałych alarmujący wydaje się odsetek przypadków o nieznanej etiologii. Trudno jest ocenić, w jakim stopniu niestabilna sytuacja ekonomiczna młodych ludzi i ich rodzin wpływa na możliwości leczenia osób powyżej 18 roku życia.

Informacje zawarte w Karcie Zgonu pozwalają na szereg pogłębionych analiz, którym mogą być poświęcone kolejne opracowania. Obecnie zasygnalizowano tylko różnice w umieralności dzieci i młodzieży związane z płcią, pomijając zupełnie wpływ miejsca i regionu zamieszkania. Celowe wydaje się również zaplanowanie pogłębionych analiz poświęconych poszczególnym klasom przyczyn zgonów oraz dodatkowych badań z retrospektywnym sięgnięciem do dokumentacji medycznej [16].

W wielu cytowanych opracowaniach podkreślano, że każdy zgon dziecka lub nastolatka powinien być traktowany jako osobista tragedia jego rodziny, jednak wnikliwa analiza poszczególnych przypadków, może służyć opracowaniu strategii poprawy opieki zdrowotnej nad dziećmi. Jak podkreślają Roberts i Jackson, oprócz analiz tzw. obciążenia chorobami, polegających na „ilościowym” monitorowaniu chorobowości i umieralności, należałoby poddawać ocenie wdrażane rozwiązania i metody postępowania medycznego w przypadku konkretnych chorób przewlekłych i o ostrym przebiegu [33]. Autorzy operują tu pojęciem podejścia opartego na rozwiązaniach (*solution-oriented apporach*). Dostępne klasyfikacje chorób i przyczyn zgonów nie dają podstaw do wnioskowania o rzeczywistych okolicznościach zgonu, w tym o czasie i rodzaju podjętej interwencji oraz współwystępujących czynnikach środowiskowych. Według Sidebothama i wsp., kluczowe znaczenie ma opracowanie krajowych procedur identyfikacji stopnia ciężkości przypadku [15].

Przyjęty w pracy okres obserwacji pozwolił na nakreślenie średniookresowych zmian w strukturze przyczyn zgonów. Za sukces należy uznać zmniejszenie udziału chorób zakaźnych i pasożytniczych w statystykach zgonów dzieci i młodzieży. Dużo trudniejsze wydaje się ograniczenie częstości występowania i skutków chorób, które mają bardziej złożone uwarunkowania i wymagają działań wielosektorowych. Ciągle trudno jest też na podstawie dostępnych źródeł danych zebrać informacje o chorobach rzadkich, które nie stanowią odrębnej klasy w istniejących systemach kodowania. Można mieć nadzieję, że wprowadzenie klasyfikacji ICD-11, częściowo rozwiąże związane z tym problemy. Należy też sądzić, że poza doniesieniami kazuistycznymi wiedzę na temat chorób rzadkich, możliwości ich diagnozowania i leczenia będzie pogłębiać współpraca międzynarodowa.
